# Liver ENPP1 protein increases with remission of type 2 diabetes after gastric bypass surgery

**DOI:** 10.1186/s12876-014-0222-x

**Published:** 2014-12-24

**Authors:** Vinko Besic, Richard S Stubbs, Mark T Hayes

**Affiliations:** Wakefield Biomedical Research Unit, Department of Pathology and Molecular Medicine, University of Otago, Wellington, New Zealand; The Wakefield Clinic, Wakefield Hospital, Wellington, New Zealand; The John Curtin School of Medical Research, The Australian National University, Canberra, Australia

## Abstract

**Background:**

Type 2 diabetes mellitus (T2DM) is a progressive disease resulting from increasing insulin resistance and reduced pancreatic β-cell insulin secretion. Ectonucleotide pyrophosphatase/phosphodiesterase 1 (ENPP1) inhibits insulin signalling and may contribute to the pathogenesis of T2DM. Others have found elevated ENPP1 levels in muscle, fat, and skin tissues from insulin resistant individuals, but similar data on liver ENPP1 is lacking. The purpose of this study was to compare expression and protein concentrations of ENPP1 in liver between patients with and without T2DM.

**Methods:**

Roux-en-Y gastric bypass surgery (RYGB) results in remission of insulin resistance and T2DM thus presenting an opportunity to examine some critical aspects of these conditions. We measured liver ENPP1 gene and protein expression in individuals with or without T2DM at RYGB and on average 17 (±5.6) months later.

**Results:**

We found liver ENPP1 protein abundance was lower in individuals with T2DM than in those with normal glucose tolerance, and increased after RYGB surgery in those individuals who had remission of T2DM. ENPP1 positively correlated with insulin sensitivity at the liver (as measured by HOMA-IR), which is contrary to what others have reported in other insulin target tissues.

**Conclusions:**

Liver ENPP1 expression in T2DM is the reverse of that expected based on expression in other tissues and is likely due to the unique role the liver has in insulin clearance. The work presented here adds another dimension to the role of ENPP1, and supports the hypothesis that ENPP1 may act as a natural modulator of insulin signalling in the liver.

**Electronic supplementary material:**

The online version of this article (doi:10.1186/s12876-014-0222-x) contains supplementary material, which is available to authorized users.

## Background

Type 2 diabetes mellitus (T2DM) is a multifactorial disease that results from increasing insulin resistance and relative loss of pancreatic β-cell function. It has reached epidemic proportions world-wide, with incidence predicted to increase from ~285 million in 2010 to ~552 million by the year 2030 [1,2]. At first used as therapy for morbid obesity, bariatric surgery can also improve T2DM and associated co-morbidities [3–5]. Although there is no clear consensus on the mechanisms that cause remission of diabetes, one common aspect among the varied bariatric surgeries is the improvement in insulin sensitivity [6,7].

Several molecules inhibit insulin signaling through the insulin receptor (INSR) and may contribute to pathogenesis of insulin resistance [8]. One of these is ectonucleotide pyrophosphatase/phosphodiesterase 1 (ENNP1) which interferes with insulin binding to the INSR α-subunit [9]. Studies have shown ENPP1 protein abundance is increased in skeletal muscle, adipose tissue and skin fibroblasts from insulin resistant individuals [10–13]. Overexpressing ENPP1 in animals led to weakened insulin signaling and action [14,15], while selectively suppressing the levels of ENPP1 in the liver of mice improved insulin sensitivity [16]. Despite the role of ENPP1 in insulin resistance, data on its role in human liver insulin resistance is lacking.

Roux-en-Y gastric bypass surgery can induce acute remission of diabetes before any substantial weight loss occurs [17–21]. Insulin resistance –as measured by HOMA-IR– decreases rapidly post RYGB surgery with improvements in diabetes [18,21,22], while peripheral insulin resistance –as measured by the euglycemic-hyperinsulinemic clamp– only improves with substantial weight loss [18,19]. As HOMA-IR is used in the fasting state, where normoglycaemia is regulated by liver glucose output, it is more indicative of liver insulin resistance. This suggests improvement of liver insulin resistance is central to remission of T2DM after RYGB surgery and provides a unique opportunity to examine the role of ENPP1 in these conditions.

In this study, our primary aim was to examine ENPP1 levels in liver tissue taken at RYGB surgery and later after remission of diabetes. We hypothesized that liver ENPP1 levels would be higher in individuals with T2DM and would decrease after remission of diabetes.

## Methods

We examined ENPP1 protein and mRNA expression using liver tissue taken from individuals who underwent open RYGB surgery as described in detail by our group elsewhere [23]. All patients having RYGB surgery for weight loss at Wakefield Hospital from 2001 to 2011 were considered for data and tissue collection. Procedures were conducted under ethical approval from the Central Health and Disability Ethics Committee of the New Zealand Ministry of Health (approval No. WGT/00/04/030). Written informed consent was given by all individuals that were included in the study and all clinical investigations were conducted according to the principles expressed in the Declaration of Helsinki. All surgeries were performed by the same surgeon (Professor Stubbs). During RYGB surgery various blood and tissue samples were taken including a liver biopsy using a Tru-Cut® Soft Tissue Biopsy Needle (Cardinal Health). A second liver biopsy was taken from most individuals who returned for further unrelated surgery.

Fifty five individuals, from a tissue bank of 448, were included in this study based on presence or absence of diabetes and availability of cryogenically stored liver tissue. The study group included those who had: normal glucose tolerance (NGT group: n = 19), impaired glucose tolerance (IGT group: n = 9), and type 2 diabetes (T2DM group: n = 27) (Table 1). Sixteen individuals of the 55 had repeat liver biopsy taken during unrelated procedures on average 17 (±5.6) months later and after significant weight loss. Of the follow up surgeries, 14 were incisional hernia repairs, while two were ring removal procedures. For comparisons at RYGB, the groups are labelled NGT, IGT, T2DM, and include the 16 patients who had repeat surgeries. Of the 16 repeat surgery individuals, 8 had diabetes which was in remission by the time of second liver biopsy and 8 had normal glucose tolerance and were insulin sensitive at RYGB surgery (Table 2). Duration of diabetes prior to its remission in individuals who had a repeat liver biopsy is as follows: diabetes was previously unrecognised in 3 patients, recently diagnosed (<1 year) in 3, and had a duration of 5 and 9 years in two others. These individuals were further classified into four repeat operation groups as follows: individuals with diabetes at RYGB surgery (rsT2DM-RYGB), individuals with remission of diabetes at operation 2 (rsT2DM-OP2), individuals with normal glucose tolerance at RYGB surgery (rsNGT-RYGB), and individuals with normal glucose tolerance at operation two (rsNGT-OP2).

**Table 1:** **Anthropometric and metabolic data of 55 obese individuals at RYGB surgery**

**Variables**	**NGT (n = 19)**	**IGT (n = 9)**	**T2DM (n = 27)**	**rsNGT (n = 8)**	**rsT2DM (n = 8)**
Age	44 ± 8	47 ± 10	54 ± 7*	43 ± 9	50 ± 7
BMI (kg/m2)	46 ± 6	49 ± 4	49 ± 10	44 ± 5	49 ± 10
HbA1c (%)	5.5 ± 0.4	5.6 ± 0.5	7.6 ± 1.1*	5.4 ± 0.2	7.1 ± 1
HOMA-IR	3.7 ± 3.6	6.1 ± 3.1	10.8 ± 8.6*	2.5 ± 0.5	10.3 ± 6.2
Fasting insulin (pmol/L)	109 ± 96	164 ± 80	196 ± 115†	80 ± 16	219 ± 127
Fasting glucose (mmol/L)	5.1 ± 0.6	5.7 ± 0.57	8.1 ± 2.3*	4.9 ± 0.4	7.4 ± 1.9
Gender					
Female (%)	16 (84)	6 (66)	15 (55)	6 (75)	5 (63)
Male	3	3	12	2	3
Diabetes					
Previously unrecognized (%)	-	-	7 (27)	-	3 (37.5)
Diet Controlled (%)	-	-	2 (8)	-	1 (12.5)
Oral hypoglycaemics (%)	-	-	14 (50)	-	4 (50)
Insulin taking (%)	-	-	4 (15)	-	0

rsNGT and rsT2DM groups represent the 16 individuals from the NGT and T2DM group respectively who had repeat liver biopsy. NGT vs T2DM data are significantly different; *p < 0.001, †p < 0.05 (ANOVA). Values are presented as mean ± SD.

**Table 2:** **Metabolic status of individuals at RYGB and operation 2**

**Variables**	**rsNGT group (n = 8)**		**rsT2DM group (n = 8)**	
	**RYGB**	**OP2**	**RYGB**	**OP2**
BMI (kg/m^2^)	44 ± 5	28 ± 4^*^	49 ± 10	30 ± 5^*^
HbA_1c_ (%)	5.4 ± 0.2	5.0 ± 0.7	7.1 ± 1	5.5 ± 0.2^†^
HOMA-IR	2.5 ± 0.45	0.78 ± 0.34^†^	10.3 ± 6.2	1.4 ± 0.8^*^
FPI (pmol/L)	80 ± 16	27 ± 13^*^	219 ± 127	48 ± 23^†^
FPG (mmol/L)	4.9 ± 0.4	4.7 ± 0.4	7.4 ± 1.9	4.7 ± 0.6^†^

RYGB vs OP2 data are significantly different *p < 0.001, ^†^p < 0.05 (Paired t-test). Values are presented as mean ± SD.

### Data collection and T2DM diagnosis

Clinical data on all patients was collected prior RYGB surgery and included: weight, height, body mass index, glycated haemoglobin (HbA1c), fasting plasma glucose, and fasting plasma insulin. Diagnosis of T2DM was established by either 1) prior documentation of diagnosis and/or receipt of treatment for T2DM or 2) diagnosis based of an oral glucose tolerance test (OGTT) routinely performed in all patients without a known history of diabetes (plasma glucose > 11.1 mmol/l at 2 hours). Impaired glucose tolerance was also diagnosed using the OGTT (plasma glucose > 7.8 and < 11.0 mmol/l at 2 hours). Individuals with T2DM were further classified as: previously unrecognized, diet controlled, needing oral hypoglycaemic drugs or insulin taking. For those individuals who had a second liver biopsy, weight, body mass index, HbA1c, fasting plasma glucose and fasting plasma insulin were measured within 2 months of the second surgery. Remission of T2DM was defined according to recommendations of Buse *et al.* (HbA1c% <6.5%, fasting plasma glucose was <5.6 mmol/l and no continuing treatment one year after surgery) [24].

### Biochemical testing

All patients undertook a 12 hour fast prior to blood collection for biochemical tests. Fasting was either self-administered or administered during the hospital stay. Clinical biochemistry testing was conducted by Aotea Pathology (Wellington, New Zealand) while testing for insulin was undertaken at Canterbury Health Laboratories (Christchurch, New Zealand).

### Estimation of insulin resistance

Homeostasis model assessment (HOMA) was used to estimate insulin resistance [25]. HOMA-IR less than 2.5 has been shown to be representative of a metabolically normal, insulin sensitive population [26].

### Liver RNA extraction

Total RNA was extracted using TRIzol® (Life Technologies) as per the manufacturer’s instructions. Briefly, 2-3 mg of frozen liver tissue was pulverized under liquid nitrogen and 800 μl of TRIzol® was added to the resultant powder which was then homogenised with an Omni Tissue Homogenizer (Omni International). 200 μl of chloroform was added to separate the mixture into a red phenol-chloroform phase, an interphase and an RNA containing colourless upper aqueous phase. The aqueous phase was collected and total RNA was precipitated with isopropyl alcohol and GlycoBlue (50 μg/mL) (Life Technologies). The resultant RNA pellet was washed with 75% ethanol, air dried and re-suspended in 20 μl DEPC-treated water. RNA was stored at −80°C prior to quality control and use in first strand cDNA synthesis. Quality and quantity of RNA was analyzed on the 2100 Agilent Bioanalyzer, using the Agilent RNA 600 Nano kit and BioSizing software (Agilent Technologies).

### cDNA synthesis and RT-qPCR

Total RNA was reverse transcribed using the SuperScript Vilo cDNA synthesis kit (Life Technologies) according to the manufacturer instructions. 1 μg of RNA was added to a 20 μl reaction mixture and the resulting cDNA was diluted 1:10 and stored at −20°C prior to use in RT-qPCR reactions. EXPRESS qPCR SuperMix (Life Technologies) and TaqMan Gene Expression Assays (ENPP1/PC-1 Hs 01054040_m1- Applied Biosystems) were used for all RT-qPCR reactions which were conducted on an ABI 7300 Real-Time PCR System (Applied Biosystems). Each sample was run in triplicate and each time a threshold cycle (Ct) was obtained using the 7300 Sequence Detection Software 1.3.1. The average of all three replicates for a particular sample was calculated and used in subsequent data analysis. Changes in gene expression were expressed as relative amount normalised to a reference gene and as fold change, both of which were calculated using the RQ method (2^-∆∆Ct^). Eukaryotic 18S rRNA (4319413E, Applied Biosystems) was used as the reference gene.

### Liver protein extraction

Protein for three of the individuals included in the study was not available for the RYBG time point. Protein was extracted by pulverizing 3-4 mg of frozen liver under liquid nitrogen. The resultant powder was incubated with buffer containing Complete Protease Inhibitor Cocktail (Roche), 30 mM TrisCl, 7 M urea, 2 M thiourea and 4% (w/v) CHAPS) for 45 minutes on ice, with vigorous vortexing every 15 minutes. The mixture was centrifuged at 14000 g for 10 minutes to remove any insoluble material. The supernatant was aliquoted for storage at −80°C. Protein was quantified using Bradford reagent (Bio Rad) on a Bio-Rad Benchmark microplate reader [27].

### E-PAGE electrophoresis and western blotting

An E-PAGE 48 well protein electrophoresis system was used to resolve protein samples on pre-cast E-PAGE 48 well 8% gels (20-300 kDa separation range) (Life Technologies) under denaturing conditions. Two separate gels were run, the first with 39 samples from the RYGB only group and the second containing 13 sets of samples from the patients who had repeat operations. Results for the RYGB comparison from the second gel were combined with the first for analysis purposes. SeeBlue Plus2 Pre-stained Standard (Life technologies) and MagicMark XP Western Protein Standard (Life technologies) were used as molecular weights to assess protein migration during electrophoresis and western blotting respectively. Protein was transferred onto Hybond-P PVDF membrane (Amersham) and blocked with 5% skim milk powder in TBS-Tween at 4°C overnight. Membranes were probed for 2 h at room temperature with monoclonal ENPP1/PC-1 (1:500, Clone F-8, Santa Cruz) [28], or monoclonal anti-actin (1:10000, Clone C4, Millipore). Blots were then incubated with alkaline phosphatase-conjugated anti mouse antibody (Life Technologies) for 0.5 h. The chemiluminescent signal was developed using CDP-Star chemiluminesent substrate (Life Technologies) and captured on x-ray film (Kodak). The protein bands on the x-ray film were digitized by the Chemidoc XRS system (Bio Rad) and then analyzed using Quantity One software (Bio Rad). Bands were quantified and expressed as volume; the *sum of the intensities of the pixels within a defined volume boundary × pixel area (intensity units × mm*^*2*^*)*. Local background subtraction was used to facilitate background correction. Protein relative abundance was calculated by normalizing the volume of the ENPP1 band to the volume of the Actin band.

### Statistical analysis

Statistical analysis was performed on 2^-ΔCt^ RT-qPCR data between each study group. Consequently gene expression data was expressed as both 2^-ΔCt^ in graphical form, whereas the fold change for significant differences was reported in text. Statistical analysis of protein abundance was performed on the value generated from normalisation of band intensity to the loading control.

Normal distribution of all groups to be compared was tested with Kolmogorov-Smirnov test. Non-normally distributed data was log transformed to comply with parametric test assumptions. Pre-post comparisons were carried out using a paired t-test. ANOVA with Bonferroni correction was used to test significance between groups. The Pearson product–moment correlation coefficient was used to test the strength and significance of association between variables. An alpha level of 0.05 was set as the significance threshold. All analysis was performed using Minitab 15. Graphical visualization was performed using GraphPad Prism 5.

## Results

### Liver ENPP1 levels at RYGB surgery

We measured levels of ENPP1 mRNA expression and protein abundance in individuals with and without T2DM and again after resolution of insulin resistance and remission of T2DM. Table 1 summarizes the relevant anthropometric data and metabolic characteristics for the study groups around the time of RYGB surgery. All individuals were morbidly obese before surgery (BMI > 40 kg/m^2^) and there were no statistically significant differences in BMI between any of the groups. The T2DM group was significantly older than the NGT group, likely owing to the chronic and progressive nature of the disease. Mean fasting plasma glucose and HbA1c levels were normal in the NGT group, and slightly raised in the IGT group. However there were no statistically significant differences between the NGT and IGT group in any of the parameters measured at RYGB surgery. The T2DM group had significantly higher (p < 0.001, ANOVA) HbA1c and fasting glucose levels than the NGT group. Although insulin resistance was present in varying severity in all groups, the T2DM group were the most insulin resistant as they had the highest mean fasting plasma insulin concentration and HOMA-IR values, which was only significantly different between the T2DM and the NGT group (p < 0.001, ANOVA).

**Figure 1:**
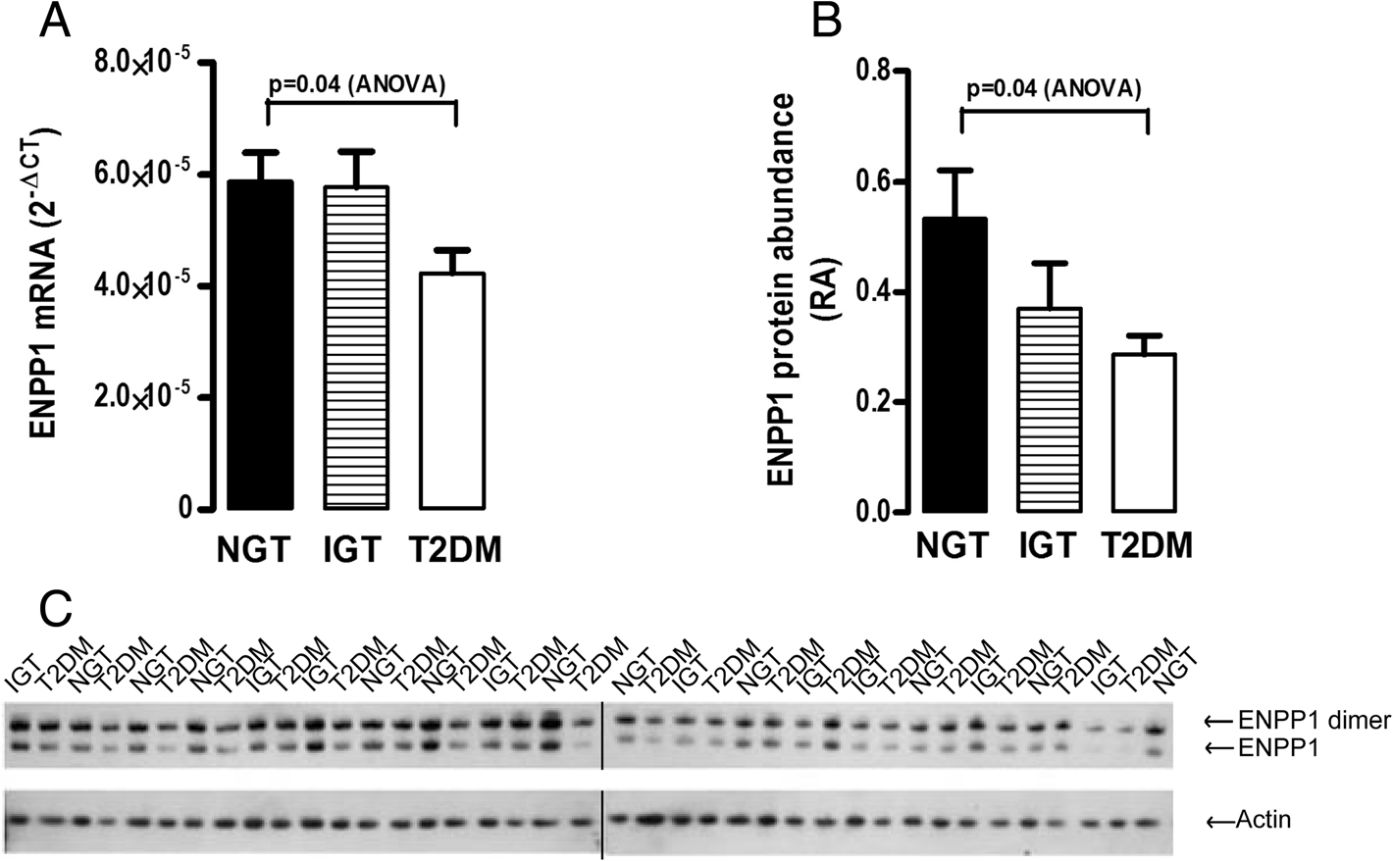
**ENPP1 gene expression and protein abundance in liver tissue of morbidly obese individuals at RYGB surgery. (A)** ENPP1 mRNA expression presented as 2^*-∆Ct*^ normalised to 18S. **(B)** ENPP1 protein abundance relative to Actin. Both ENPP1 mRNA and protein abundance was significantly lower in the T2DM group in comparison to the NGT group. **(C)** ENPP1 western blot from liver of 39 individuals who did not have a second liver biopsy. The remaining 13 samples were run on a different gel (Figure 3B) so as to facilitate easier comparison of ENPP1 protein before and after remission of diabetes and insulin resistance. The data from the two gels was combined for analysis of samples at RYGB. Black lines denote non-adjacent lanes. For uncropped blots see additional document 1. NGT; Normal glucose tolerance (n = 19), IGT; Impaired glucose tolerance (n = 9), T2DM; Type 2 diabetes (n = 27). Data is presented as mean + SE.

We found that liver ENPP1 gene expression was −1.5 (95% CI −6.2 to 2.8) fold lower in the T2DM group when compared to the NGT group (Figure 1A, p = 0.04, ANOVA), while there was no difference in ENPP1 mRNA expression between the NGT and IGT group. Figure 1C displays the western blot for ENPP1 protein abundance only for the 39 individuals who did not have a second liver biopsy (See Additional file 1 - Uncropped Western Blots, Figures 1 (ENPP1) & 3 (Actin) for uncropped original blots). To avoid gel bias, sample loading was alternated based on disease state. The molecular weight of ENPP1 in SDS-PAGE is 110–130 kd depending on tissue type. Some proportion of ENPP1 reportedly exists as dimer and the 220kd band visible in Figure 1C is most likely the dimeric form of ENPP1 [29]. The liver ENPP1 protein abundance was −2 fold lower (95% CI, −3.6 to −1.3) in the T2DM group in comparison to the NGT group (Figure 1B), which was also statistically significant (p = 0.04, t-test). To control for the effect of treatment on ENPP1 levels, we performed ANOVA analysis to test whether different treatments for diabetes affected ENPP1 levels. There was no significant differences in ENPP1 mRNA (p = 0.221) or ENPP1 protein (0.362) between previously unrecognized, diet controlled, insulin taking, or oral hypoglycemic taking individuals. Although ENPP1 protein abundance was lower in the IGT group, it did not reach statistical significance. Retrospective power calculations show that while the sample sizes in the NGT and T2DM are sufficient to reject the null hypothesis (α-level 0.05, power 0.83), the small sample size in the IGT group is limiting and another 36 observations would be needed to reach a statistically significant result.

**Figure 2:**
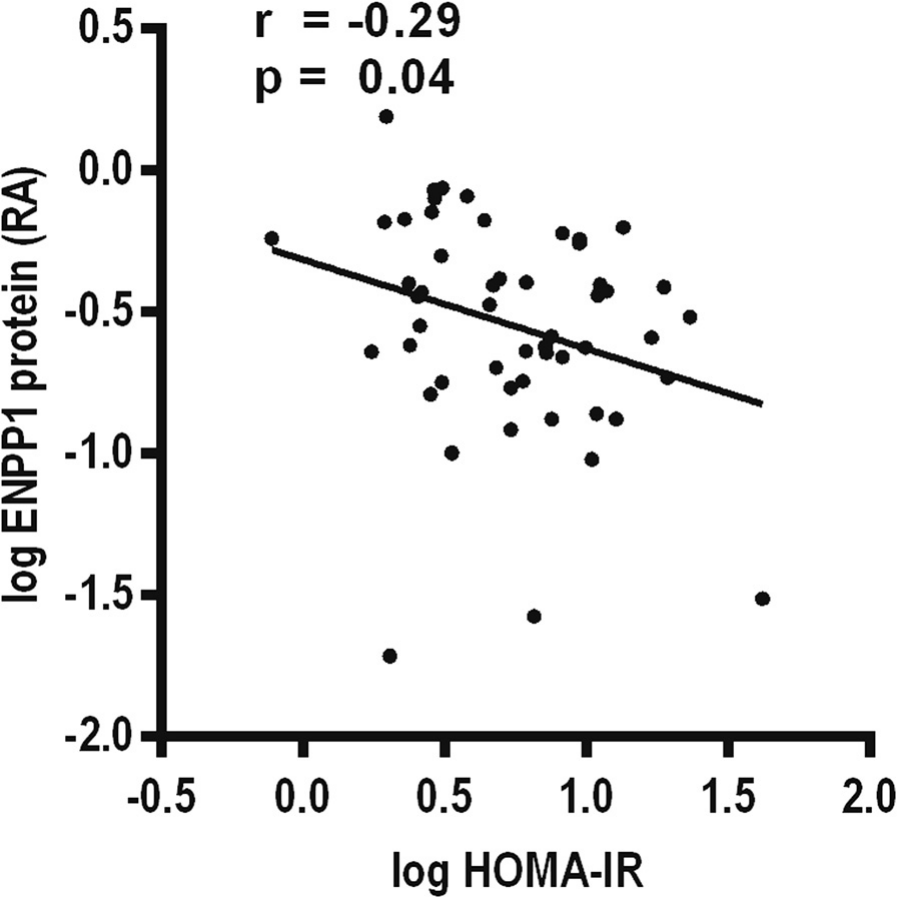
**ENPP1 relationship with liver insulin sensitivity.** Pearson product–moment correlation of liver ENPP1 protein relative abundance (log) vs HOMA-IR (log) (n = 52).

To explore any relationship between liver ENPP1 protein and insulin sensitivity, we calculated a Pearson product–moment correlation between ENPP1 and HOMA-IR. ENPP1 protein abundance negatively correlated with HOMA-IR in the whole cohort (Figure 2), suggesting a positive association between ENPP1 and insulin sensitivity.

### Liver ENPP1 levels before and after RYGB surgery

One of the novel characteristics of this study is the use of liver tissue biopsied from the same individual before and after significant weight loss and improvement in insulin sensitivity and/or remission of type 2 diabetes. Liver ENPP1 mRNA expression and protein abundance was measured in individuals who had normal glucose tolerance (rsNGT group) or T2DM (rsT2DM group) at RYGB and at a second operation 17 months later. Table 2 lists the metabolic characteristic for the rsNGT and rsT2DM groups at first and second liver biopsy. Out of the 16 individuals who had a second liver biopsy, two had incomplete follow up data. Both the rsNGT and rsT2DM groups had significant decreases in BMI by the second operation. There were no statistically significant differences in BMI between the two groups at either operation or in amount of weight lost, thus removing weight loss as a potential confounding factor. The rsT2DM group had a significant (p < 0.05, paired t-test) decrease in mean fasting plasma glucose and HbA1c levels, representing remission of T2DM. Liver insulin sensitivity (as measured by HOMA-IR) improved dramatically in both the rsNGT and rsT2DM group. The rsT2DM group had the greatest improvement in insulin sensitivity which coincided with remission of diabetes.

**Figure 3:**
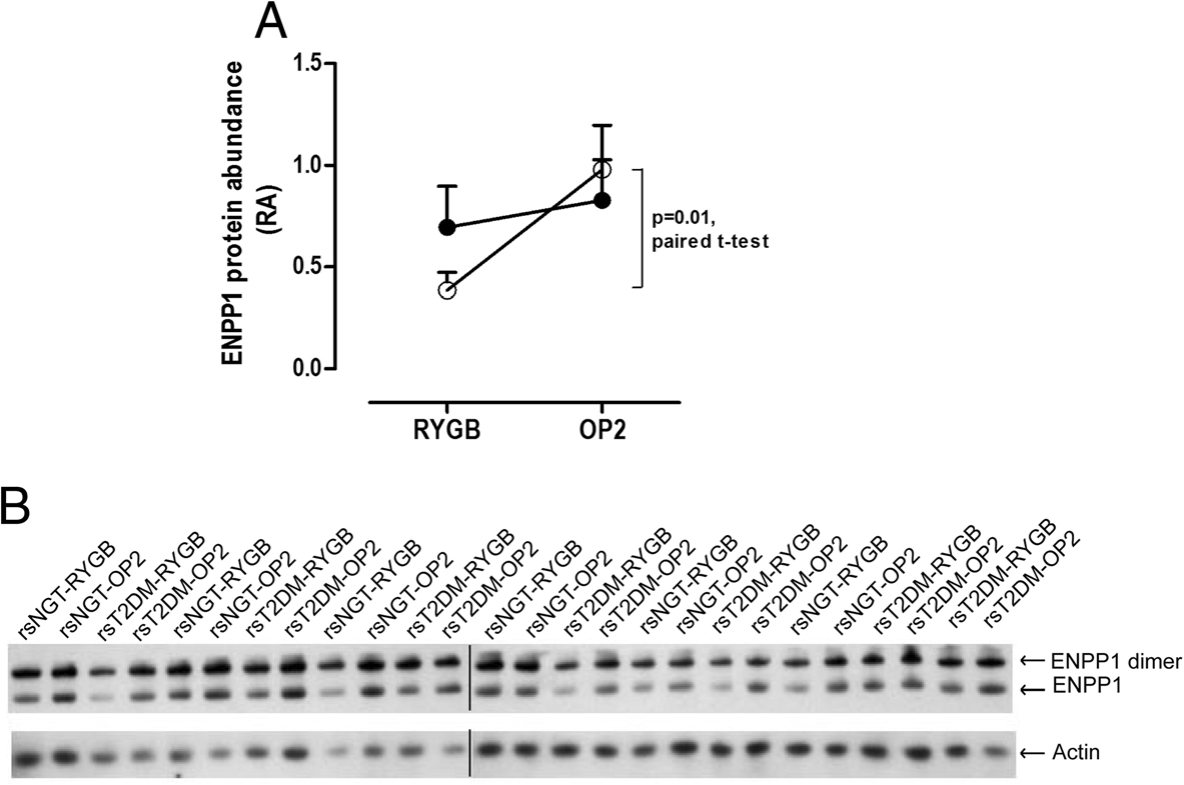
**Liver ENPP1 protein abundance at RYGB surgery and at operation 2. (A)** ENPP1 protein abundance had a significant increase in the rsT2DM group after RYGB surgery. **(B)** Corresponding western blot for ENPP1 for individuals that had a second liver biopsy. Black lines denote non-adjacent lanes. For uncropped blots see additional document 1. rsNGT ●: repeat surgery normal glucose tolerance (n = 6); rsT2DM ○: repeat surgery type 2 diabetes (n = 7). Data is presented as mean + SE.

Figure 3A shows the mean liver ENPP1 protein abundance for all 13 individuals who had liver protein extracted at RYGB surgery and on average 17 months later, while Figure 3B is the corresponding western blot (see Additional file 1 - Uncropped Western Blots - Figures 2 (ENPP1) and 4 (Actin) for original uncropped images). There were six pairs of liver from the rsNGT group while there were seven pairs of samples from the rsT2DM group. Although the rsNGT group had no great change in liver ENPP1 protein levels after RYGB surgery, the rsT2DM group had a statistically significant (p = 0.01, paired t-test) increase in ENPP1 protein abundance (Figure 3A). The individuals who had resolution of insulin resistance and remission of T2DM following RYGB had a mean 2.7 (95% CI, 1.8 to 4.0) fold increase in liver ENPP1 protein abundance. Thus, improvement in liver insulin sensitivity after RYGB surgery as measured by HOMA-IR was concomitant with increasing levels of ENPP1 protein at the liver in humans. Neither group had significant changes in liver ENPP1 gene expression after RYGB surgery (data not shown), suggesting that post translational regulation is more important in the regulation of ENPP1 protein level with change in insulin resistance and remission of diabetes.

## Discussion

ENPP1 may contribute to insulin resistance through its inhibitory action on insulin signalling. Although others have studied ENPP1 in various human tissues (skin, muscle, and adipose tissue) [10–12], relatively little is known about ENPP1 levels in human liver. Improvement in liver insulin resistance is associated with early improvements in glycaemic control post RYGB surgery. This occurs before significant weight loss and associated improvements in insulin resistance in fat and muscle, suggesting that the liver may be a critical component of the metabolic changes seen post-surgery [18–22]. For this reason we used liver tissue taken at RYGB surgery to examine the role of ENPP1 in liver insulin resistance. RYGB surgery improved liver insulin sensitivity –as measured by HOMA-IR– regardless of diabetes status, while remission of T2DM was associated with normalisation of fasting plasma insulin concentrations and HOMA-IR values.

From the current literature on ENPP1 we expected that its levels would be high in individuals with T2DM and decrease after remission of the disease. Surprisingly, liver ENPP1 mRNA and protein was lower in individuals with T2DM in comparison to individuals with normal glucose tolerance (Figure 1A and B) at RYGB. In the subset of individuals who had a second liver biopsy, only those that had remission of diabetes and improvement in insulin sensitivity had an increase in liver ENPP1 protein abundance (Figure 3A).

However, ENPP1 mRNA expression did not change significantly after remission of diabetes, even though individuals with T2DM had lower expression than those with normal glucose tolerance at RYGB surgery. This is likely caused by post transcriptional processing as insulin has been shown to affect liver ENPP1 protein abundance without changing its gene expression [30], which is in agreement with data presented here. Individuals with impaired glucose tolerance also tended to have decreased liver ENPP1 protein, and the pattern of liver ENPP1 protein abundance in the IGT and T2DM groups is likely caused by high circulating levels of insulin as both groups had moderate to severe insulin resistance respectively. In this study there was a significant association between increased liver ENPP1 protein abundance and increased liver insulin sensitivity (Figure 2).

Although our data do not agree with studies that have associated increased ENPP1 content in muscle and adipose tissue with insulin resistance [10–13], there is conflicting evidence as to ENPP1’s role in the pathology of diabetes. Pender *et al.* have shown no change in muscle ENPP1 content in morbidly obese humans after bariatric surgery where there had been a measured increase in insulin sensitivity [31]. In a Zucker diabetic fatty rat model of T2DM there was no difference in muscle and liver ENPP1 content in comparison to control animals [29]. Even though some experimentally produced T2DM animal models had increased ENPP1 content [32], others did not [33,34].

We suspect that the conflicting evidence reported in the literature and our own data presented here is due to the varied roles muscle, fat, and liver tissues have in regulating insulin action. The liver has a crucial role in regulating the systemic insulin concentration by hepatic insulin clearance [35]. It is the first organ to receive large amounts of insulin released from the pancreas during the first phase response (1000–5000 pmol/l) and removes ~50% of it from the blood stream [36,37]. Considering the liver extracts and recycles supraphysiological levels of insulin during first phase insulin secretion, preventing overstimulation of metabolic pathways may be necessary for normal glucose homeostasis. In line with this theory, individuals with insulin receptor or IRS-1 mutations resulting in defunct insulin signalling have low ENPP1 activity and content [38]. Menzaghi *et al.* have suggested ENPP1 may act as a natural desensitiser of insulin signalling because of insulin’s ability to stimulate ENPP1 post-translational processing and recruitment to the plasma cell membrane [30]. Our data presented here is in accordance with this hypothesis.

However, there are some limitations to the data presented here. Stefanovic *et al.* reported previously that metformin treatment can influence ENPP1 activity in lymphocytes from individuals with diabetes [39], while half of the individuals with diabetes in our study were on oral hypoglycemics (metformin). Nonetheless, we found no significant differences in ENPP1 mRNA or protein levels between different treatments for diabetes, including metformin, which is in agreement with that of Ludovico *et al.* who have also shown that metformin does not affect ENPP1 mRNA levels in lymphocytes [40]. Repeat human liver samples are exceedingly hard to obtain and consequently there has been little published on the changes in liver gene and protein expression associated with the remission of diabetes post-RYGB. The small sample size in the IGT group limits interpretation of this data, but our study has sufficient power when comparing the NGT and T2DM groups to suggest that ENPP1 may not contribute to insulin resistance at the liver but is a normal biological regulator of insulin action in an organ which is exposed to very high levels of insulin.

## Conclusions

In this study, we have shown a reverse ENPP1 pattern to that previously described in muscle and adipose tissue of insulin resistant individuals. Liver ENPP1 protein abundance was lower in the liver of individuals with T2DM in comparison to individuals with normal glucose tolerance and increased after remission of T2DM. We believe this is likely due to the role of the liver in insulin processing, and our data is in agreement with the previously supposed hypothesis that ENPP1 is a natural regulator of insulin signalling strength. The decreased levels of liver ENPP1 during hyperglycaemic conditions such as diabetes may be a compensatory response to increase insulin signalling and regain control of glucose homeostasis. Although our work does not discount the possibility that ENPP1 contributes to insulin resistance, it does add another dimension that will have to be considered in future work exploring the influence ENPP1 has on liver insulin signalling.

## Additional file

Additional file 1:
**Additional Document: Uncropped Western Blots.**

## Abbreviations

T2DM: Type 2 diabetes
ENPP1: Ectonucleotide pyrophosphatase/phosphodiesterase 1
RYGB: Roux-en-Y gastric bypass surgery
T2DM group: individuals with type 2 diabetes
NGT group: individuals with normal glucose tolerance
rsNGT: individuals with normal glucose tolerance who had repeat liver biopsy
rsT2DM: individuals with type 2 diabetes who had repeat liver biopsy
OGTT: Oral glucose tolerance test
BMI: Body mass index
HOMA: Homeostasis model assessment
RT-qPCR: Real time – quantitative polymerase chain reaction
SDS-PAGE: Sodium dodecyl sulphate- polyacrylamide gel electrophoresis
