# Supplementary material for: Liver ENPP1 protein increases with remission of type 2 diabetes after gastric bypass surgery
Source: BMC Gastroenterol. 2014 Dec 24;14:222. doi: 10.1186/s12876-014-0222-x (PMC4296549; doi:10.1186/s12876-014-0222-x)
Supplement: Additional file 1: — Additional Document: Uncropped Western Blots. [file 12876_2014_222_MOESM1_ESM.pdf]

Additional Document: Uncropped Western Blots

For all uncropped western blots see Figures 1 to 4 below. To avoid gel artefacts all samples to be compared were loaded onto the minimum amount of gels. Two different gels were run per antibody. For the comparisons at RYGB surgery, individuals without type 2 diabetes were loaded adjacent to individuals with type 2 diabetes. This alternating sequence was followed through the gel.

For the pre and post RYGB comparison there were six pairs of liver samples from individuals with normal glucose tolerance (rsNGT) while there were seven pairs of livers from individuals with type 2 diabetes (rsT2DM). Pre and post RYGB samples were loaded adjacent to each other. rsNGT and rsT2DM samples were alternated.

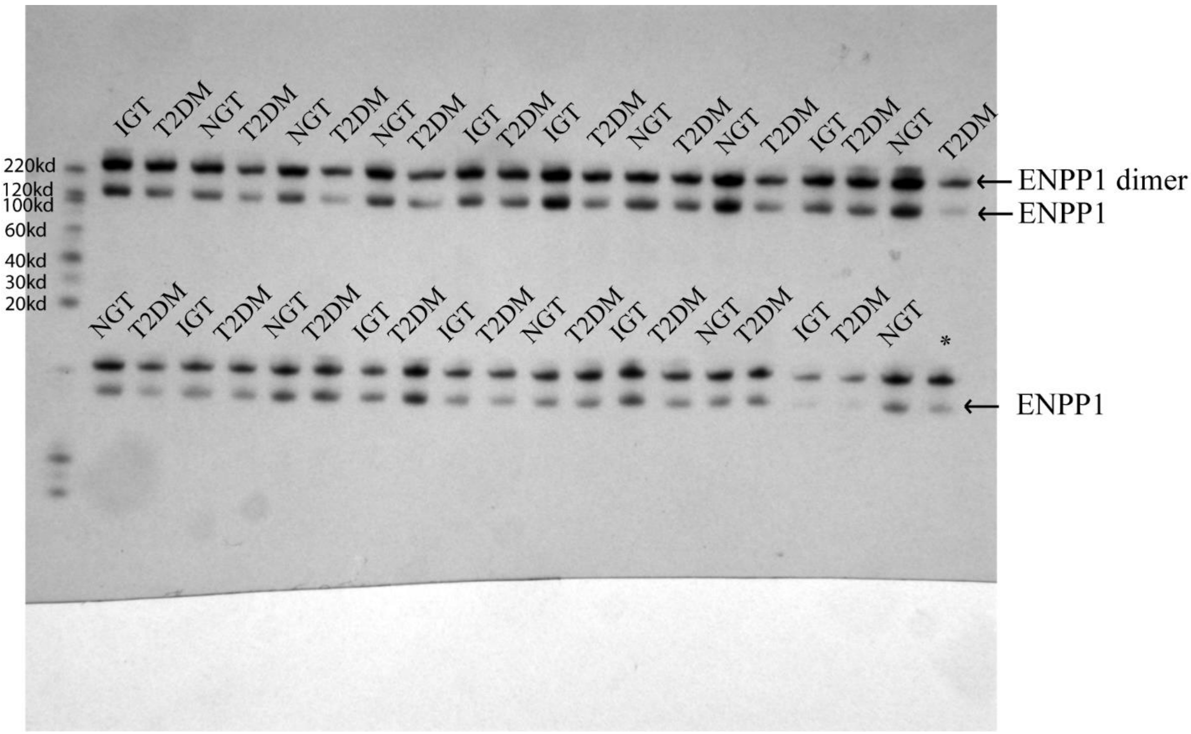

Figure 1. ENPP1 (110-130kda) western blot using protein extracted from liver tissue biopsied from individuals at the time of RYGB surgery. The lane marked with an * contained a duplicate sample.

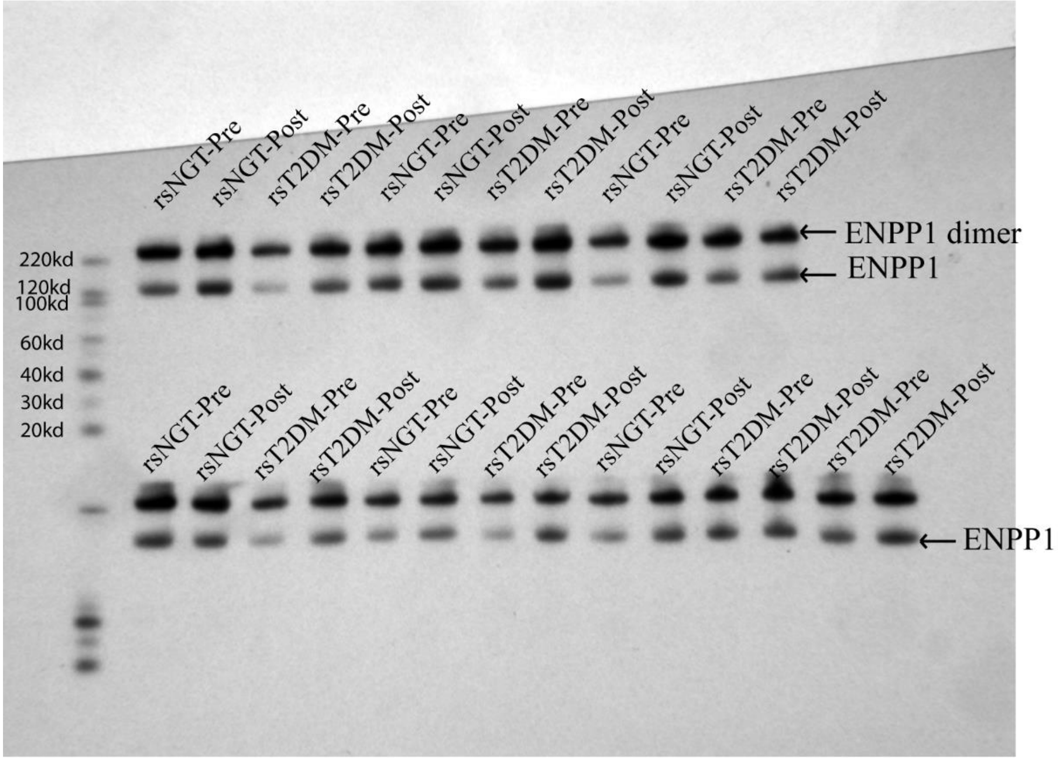

Figure 2. ENPP1 (110-130kda) western blot using protein extracted from liver tissue biopsied from individuals both at the time of RYGB surgery and again at a subsequent operation.

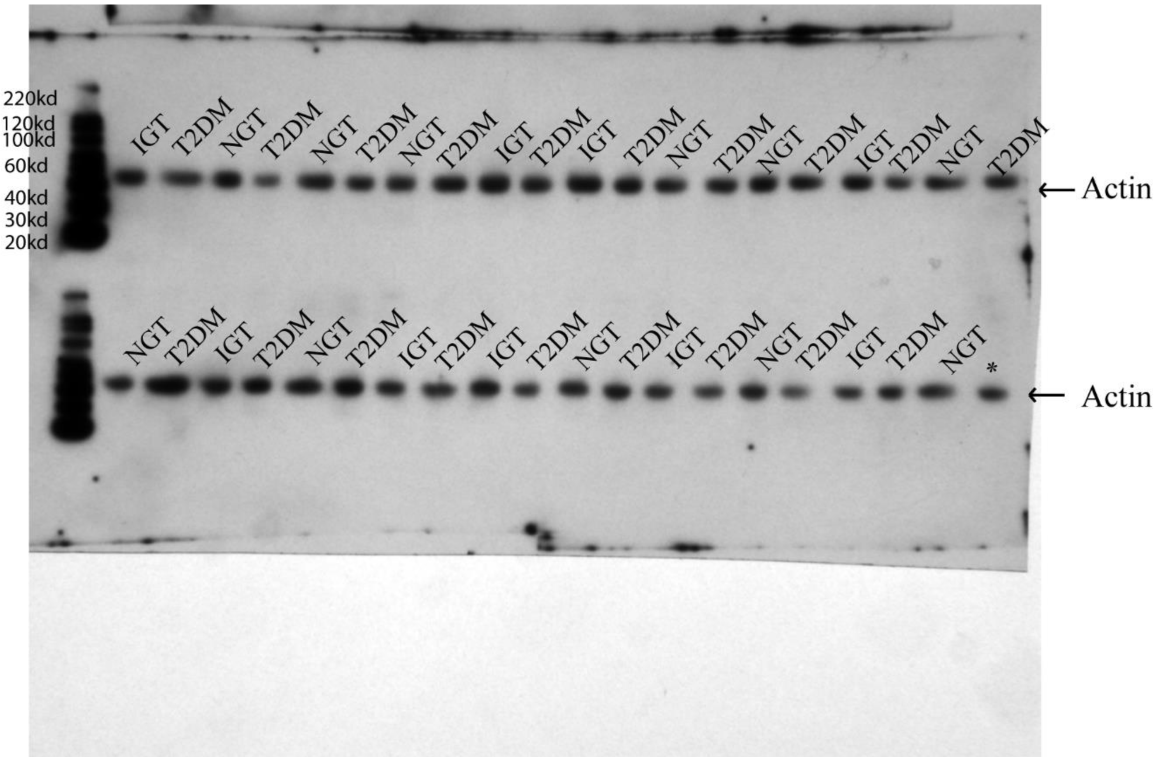

Figure 3. Actin (43kda) western blot using protein extracted from liver tissue biopsied from individuals at the time of RYGB surgery. The lane marked with an * contained a duplicate sample.

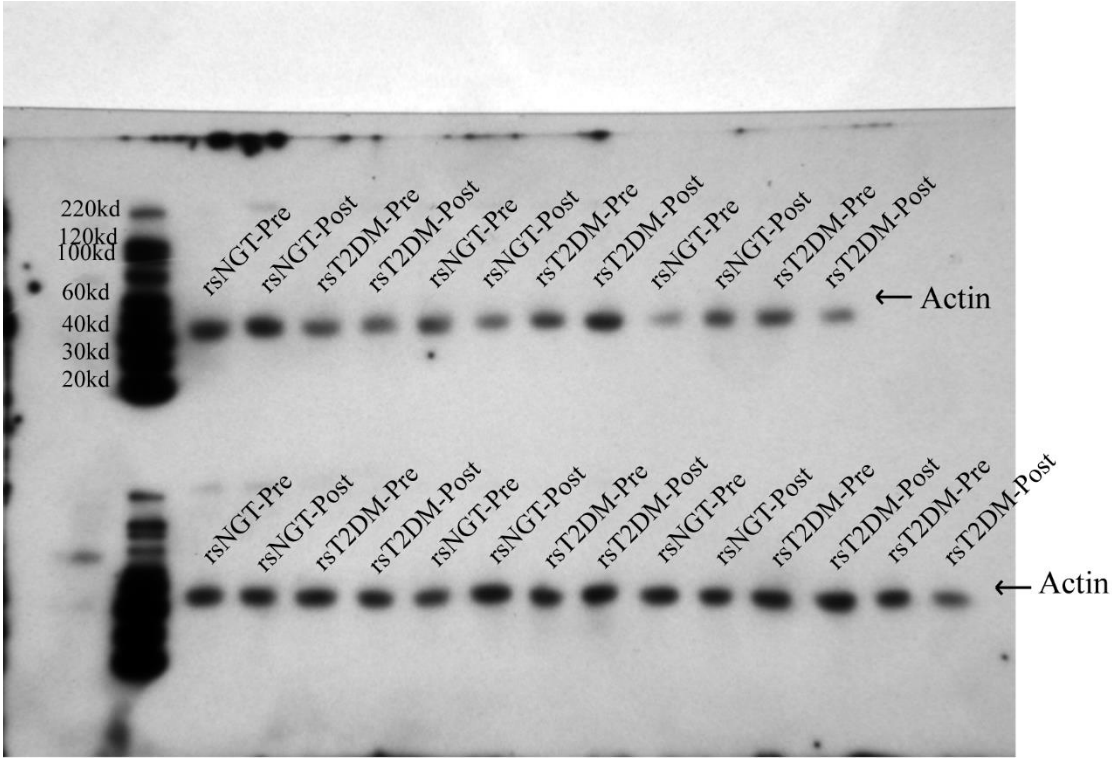

Figure 4. Actin-1 (43kda) western blot using protein extracted from liver tissue biopsied from individuals both at the time of RYGB surgery and again at a subsequent operation.
